# Roux-en-Y gastric bypass-induced bacterial perturbation contributes to altered host-bacterial co-metabolic phenotype

**DOI:** 10.1186/s40168-021-01086-x

**Published:** 2021-06-14

**Authors:** Jia V. Li, Hutan Ashrafian, Magali Sarafian, Daniel Homola, Laura Rushton, Grace Barker, Paula Momo Cabrera, Matthew R. Lewis, Ara Darzi, Edward Lin, Nana Adwoa Gletsu-Miller, Stephen L. Atkin, Thozhukat Sathyapalan, Nigel J. Gooderham, Jeremy K. Nicholson, Julian R. Marchesi, Thanos Athanasiou, Elaine Holmes

**Affiliations:** 1 Division of Digestive Disease, Department of Metabolism, Digestion and Reproduction, Imperial College London, London, SW7 2AZ UK; 2 Division of Surgery, Department of Surgery and Cancer, Imperial College London, London, SW7 2AZ UK; 3 Division of Systems Medicine, Department of Metabolism, Digestion and Reproduction, Imperial College London, London, SW7 2AZ UK; 4 Division of General and Gastrointestinal Surgery, Department of Surgery, Emory University School of Medicine, Atlanta, Georgia 30322 USA; 5 Department of Applied Health Science, School of Public Health, Indiana University Bloomington, 1025 E 7th Street, Bloomington, IN 47405 USA; 6 RCSI Bahrain, Adiya, Kingdom of Bahrain; 7 Department of Academic Endocrinology, Diabetes and Metabolism, Hull-York Medical School, Hull, UK; 8 Centre for Computational and Systems Medicine, The Health Futures Institute, Murdoch University, Harry Perkins Building, Perth, WA 6150 Australia

**Keywords:** Bariatric surgery, Bile acids, Host-microbial metabolism, Metabolic profiling, Microbiome

## Abstract

**Background:**

Bariatric surgery, used to achieve effective weight loss in individuals with severe obesity, modifies the gut microbiota and systemic metabolism in both humans and animal models. The aim of the current study was to understand better the metabolic functions of the altered gut microbiome by conducting deep phenotyping of bariatric surgery patients and bacterial culturing to investigate causality of the metabolic observations.

**Methods:**

Three bariatric cohorts (*n* = 84, *n* = 14 and *n* = 9) with patients who had undergone Roux-en-Y gastric bypass (RYGB), sleeve gastrectomy (SG) or laparoscopic gastric banding (LGB), respectively, were enrolled. Metabolic and 16S rRNA bacterial profiles were compared between pre- and post-surgery. Faeces from RYGB patients and bacterial isolates were cultured to experimentally associate the observed metabolic changes in biofluids with the altered gut microbiome.

**Results:**

Compared to SG and LGB, RYGB induced the greatest weight loss and most profound metabolic and bacterial changes. RYGB patients showed increased aromatic amino acids-based host-bacterial co-metabolism, resulting in increased urinary excretion of 4-hydroxyphenylacetate, phenylacetylglutamine, 4-cresyl sulphate and indoxyl sulphate, and increased faecal excretion of tyramine and phenylacetate. Bacterial degradation of choline was increased as evidenced by altered urinary trimethylamine-*N*-oxide and dimethylamine excretion and faecal concentrations of dimethylamine. RYGB patients’ bacteria had a greater capacity to produce tyramine from tyrosine, phenylalanine to phenylacetate and tryptophan to indole and tryptamine, compared to the microbiota from non-surgery, normal weight individuals. 3-Hydroxydicarboxylic acid metabolism and urinary excretion of primary bile acids, serum BCAAs and dimethyl sulfone were also perturbed following bariatric surgery.

**Conclusion:**

Altered bacterial composition and metabolism contribute to metabolic observations in biofluids of patients following RYGB surgery. The impact of these changes on the functional clinical outcomes requires further investigation.

**Video abstract**

**Supplementary Information:**

The online version contains supplementary material available at 10.1186/s40168-021-01086-x.

## Introduction

Obesity affects almost half a billion adults worldwide and almost one-third of children under 11 years old in Europe, and is a risk factor for cardiovascular events, type 2 diabetes, non-alcoholic fatty liver disease and some cancers [1]. Bariatric surgery is currently the most effective strategy for achieving sustained weight loss in patients who have severe obesity and refers to a suite of surgical procedures that are carried out to achieve sustained weight loss where diet and exercise programs have failed. Bariatric procedures include primarily restrictive operations that limit food intake such as laparoscopic gastric banding (LGB) and sleeve gastrectomy (SG) and restrictive/malabsorptive procedures such as Roux-en-Y gastric bypass (RYGB). Although weight loss is the primary aim, bariatric surgery has additional beneficial effects including the resolution of type 2 diabetes, hypertension and obstructive sleep apnoea [2]. However, the procedure carries surgical risk and is associated with long-term requirement for dietary supplementation. Therefore, improved mechanistic understanding of how bariatric surgery achieves sustained weight loss may open doors to less invasive solutions.

RYGB surgery has a profound metabolic effects, including increased glucose tolerance, insulin sensitivity [3] and secretions of intestinal hormones (e.g. glucagon-like peptide-1 (GLP-1), peptide YY (PYY)) [4]. The complexity of engaging multiple metabolic pathways through the post-operative period has highlighted the strengths of a systems biology approach to assess the underlying biological actions of these operations. Several studies have applied metabolic phenotyping of biofluids and tissue samples to probe the mechanisms associated with weight loss and/or resolution of type 2 diabetes but these studies have tended to be underpowered, typically including between 2 and 50 participants (Table S1) and have mainly focused on the serum or plasma metabolome. Few studies have integrated the effects of surgery on the metabolome and gut microbiome in a systematic manner. There are consistent metabolic effects across the studies suggestive of perturbations in glycine, serine and threonine metabolism; nitrogen metabolism; phenylalanine metabolism; cysteine and methionine metabolism; tricarboxylic acid cycle; branched chain amino acid (BCAA) metabolism; taurine and hypotaurine metabolism, propanoate metabolism and nicotinate; and nicotinamide metabolism (detailed in Table S1). Several of these perturbed metabolite classes and pathways involve co-regulation by the gut bacteria. Bariatric surgery exerts a profound effect on members of the gut microbiome altering the gut bacterial composition from Firmicutes and Bacteroides-dominant to Proteobacteria-dominant, particularly bacteria from *Enterobacteriaceae* family, in both human [5, 6] and rodent models [7, 8]. This observation is consistent with observed changes in faecal and urinary host-bacterial co-metabolites post-surgery, such as increased faecal putrescine, gamma aminobutyric acid (GABA) and trimethylamine (TMA) and increased urinary trimethylamine-*N*-oxide (TMAO), 4-cresyl sulphate, phenylacetylglycine/phenylacetylglutamine and hippurate [5, 7]. In addition, increasing evidence shows that bile acids (BAs) could play a role in the mechanisms of bariatric surgery. For example, BAs promote GLP-1 secretion from enteroendocrine cells and activate G-protein coupled receptor 5 (GPR5) to drive energy expenditure [9, 10]. Most of the studies have reported bile acid composition in blood; however, it is unclear how bariatric surgery influences urinary excretion of bile acids.

None of the studies so far have investigated the integrated surgery-induced impact at a systems level including both excretory (urine and faecal) and circulating (plasma) metabolites with gut bacterial composition and metabolic activity following bariatric surgery. In the current study, we studied three cohorts of patients, two from the UK and one from the USA, with a focus on the UK cohort for deep phenotyping using both proton nuclear magnetic resonance (NMR) spectroscopy and liquid chromatography mass spectrometry (LC-MS), in combination with metataxonomics and culture-based techniques to explore causality of the metabolic observations following RYGB surgery.

## Materials and methods

### Study design and sample collection

Three cohorts of patients who had undergone laproscopic bariatric surgery were included. *Cohort 1*: a total of 84 patients recruited from the Charing Cross and Hammersmith Hospitals (London, UK) under the ethical permission—Metabolic Phenotype and Modulation in Obese and Bariatric Surgical Patients 08/H0711/123. Patients underwent RYGB (*n* = 68), sleeve gastrectomy (SG; *n* = 10) or laparoscopic gastric banding (LGB; *n* = 6) respectively. Urine and faecal samples were collected at 1 month prior to the surgery, 2–6 months (short-term follow-up, SF) and 1–2 years (long-term follow-up, LF) post-surgery. Clinical data were recorded when the sample collection was carried out in the hospital. *Cohort 2*: Fourteen patients were recruited from Purdue University, USA (IRB #333-2002). Five patients underwent LGB and nine received RYGB surgery. Urinary and serum samples were collected prior to surgery and 6 months post operation for metabolic phenotyping. *Cohort 3*: Nine patients were recruited from East Yorkshire and North Lincolnshire Research Ethics Committee (ethics number: NRES 10/H1304/13 approved 09/07/2010) and underwent RYGB surgery. Blood serum samples were collected at multiple time points, including pre-op, 1-, 3-, 6-, 9- and 12-months post-operation. All samples were stored at − 80 °C until analysis. The ratio of women to men was 2.7:1, with a mean age of 43.1 years of age and a mean pre-surgery BMI of 47.52 kg/m^2^ across all studies (demographics are provided in Table S2).

**Fig. 1:**
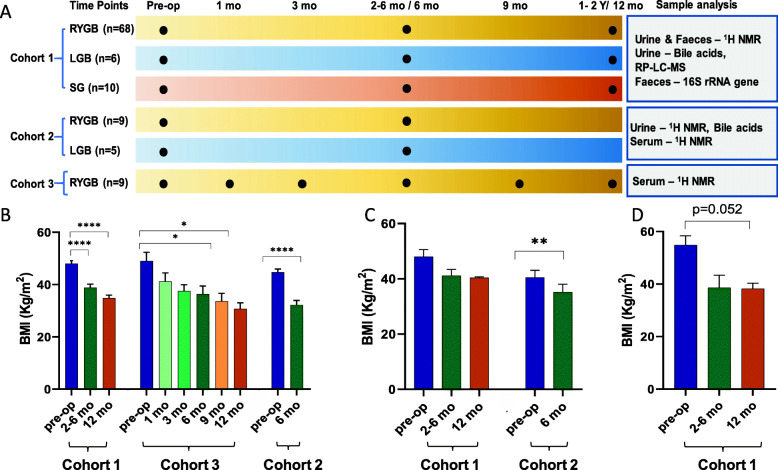
Experimental design of three cohorts of bariatric surgery patients including sampling and the applied analytical methods (**A**). Body mass index (BMI) of patients who had undergone RYGB (**B**), gastric banding (**C**) and sleeve gastrectomy (**D**) at pre-op and post-operation time points. ****p < 0.0001, **p < 0.005, *p < 0.05. Data are presented in mean ± SEM

^1^H NMR spectroscopy- and bile acid profiling analysis were carried out on all studies where samples were available and untargeted LC-MS-based metabolic profiling was performed on samples from cohort 1 (see Fig. 1A for experimental overview) according to standard analytical protocols shown in Supplementary Information. Faecal 16S rRNA gene sequencing-based bacterial compositional analysis was also carried out for cohort 1. Details of sample and data analysis are provided in Supplementary Information.

## Results

### Effect of surgery on weight loss

Weight loss was achieved by bariatric surgery in all cohorts (Table S2). Amongst the three types of bariatric surgery, RYGB surgery induced the most profound weight loss in all cohorts (Fig. 1B). A trend in weight loss induced by LGB was observed for cohort 1 but was not statistically significant and the reduction of BMI in cohort 3 was significant at 6-month post-op compared to pre-op (*p* = 0.005) (Fig. 1C). Most patients who had undergone SG achieved substantial weight loss between pre-op and LF but greater variation in weight reduction within this cohort resulted in only marginal significance (*p* = 0.052, Fig. 1D).

### Bariatric surgery profoundly alters the urinary metabolome

**Fig. 2:**
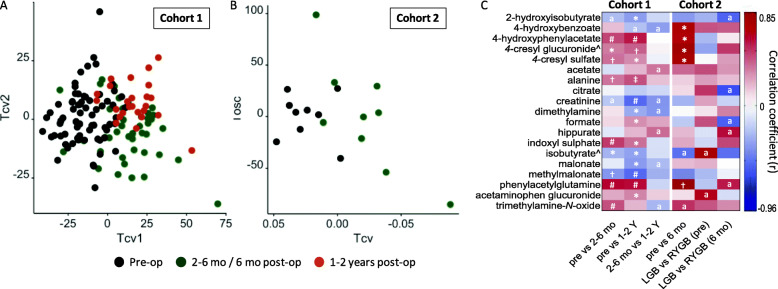
OPLS-DA cross-validated scores plots of urinary ^1^H NMR spectra of the RYGB patients from cohort 1 at pre-op, 2–6 months and 1–2 years post-op (**A**, Q^2^Y = 0.27; R^2^X = 17.2%; R^2^Y = 56.3%, CVANOVA p = 2.04 × 10^−15^), and cohort 2 at pre-op and 6-month post-op (B, Q^2^Y = 0.46; R^2^X = 25.3%; R^2^Y = 97.0%, *p* = 0.02). The metabolites that significantly contributed to the classification of different time points are shown in the heatmap (**C**). The correlation coefficient (*r*) was derived from OPLS-DA models and a positive correlation indicates higher relative concentrations of the metabolites in post-op compared to pre-op or in RYGB compared to LGB. q, the corrected *p* values using Benjamini-Hochberg multiple test correction are shown as #*q* < 0.0001, ‡*q* < 0.001, †*q* < 0.005, *q < 0.05. ^a^*p* < 0.05 and q>0.05. ^^^ putatively assigned metabolites

As with weight loss, RYGB surgery induced the largest changes in the composition of the urinary metabolome compared to SG and LGB surgeries for both cohorts 1 and 2 in terms of both the number of metabolites significantly altered between pre-op and post-op time points and the magnitude of change (Fig. 2A, B, ROC analysis of cross-validated scores is shown in Figure S1). These changes included significantly elevated concentrations of host-bacterial co-metabolites, such as 4-hydroxylphenylacetate, phenylacetylglutamine (PAG), 4-cresyl sulphate and 4-cresyl glucuronide in both cohorts post-surgery (Fig. 2C). The urinary level of 4-hydroxybenzoate was significantly higher post-op in cohort 2 but not cohort 1. Increased urinary excretion of the gut bacterial-host co-metabolites indoxyl sulphate and trimethylamine *N*-oxide (TMAO) after RYGB surgery was observed in both cohorts 1 and 2 but was only significant for cohort 1. Although there was a trend towards increased excretion of bacterial metabolites post-operation for LGB or SG operations, no urine metabolites were statistically significant between pre- and post-op. The difference in the magnitude of effect between RYGB and LGB is further emphasized by the fact that significantly higher levels of host-bacterial co-metabolites were noted in participants undergoing RYGB compared to LGB (Figure S2).

Whilst urinary creatinine levels decreased in cohorts 1 and 2 post-RYGB, reaching a statistical significance at LF, urinary levels of hydroxymyristic acid and 3-hydroxydicarboxylic acids (e.g. 3-hydroxytetradecanedioic acid, 3-hydroxydodecanedioic acid) increased following RYGB surgery (Figure S3). Acetaminophen glucuronide was found to be higher in RYGB patients post-op with a statistical significance at LF, suggesting a higher intake of acetaminophen following RYGB. Literature reports stating that 94% of patients undergoing RYGB surgery had recurring abdominal pain 5 years after surgery [11]. It is possible that the observed increase in acetaminophen metabolites may be a response to prolonged surgery-associated pain.

**Fig. 3:**
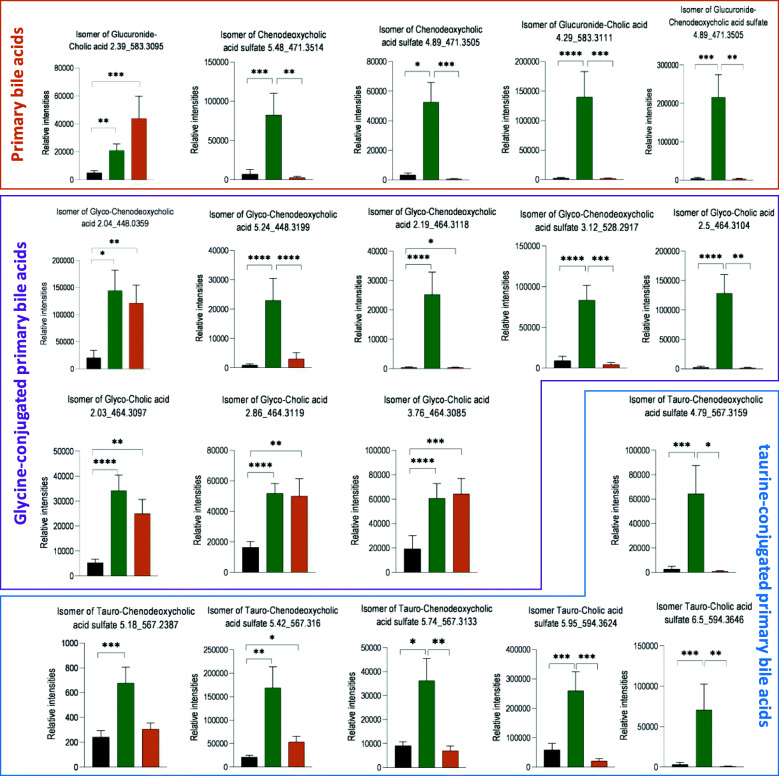
Relative intensities of urinary bile acids from cohort 1 RYGB patients at pre-op (black), 2–6 months (green) and 1–2 years post-op (orange). Kruskal-Wallis test was used for group comparisons and Dunn’s test was used for adjusting multiple comparisons. The adjusted *p* values: ****p < 0.0001, ***p < 0.001, **p < 0.01, *p < 0.05. Data are presented in mean ± SEM. The bile acids and their retention times (min) and m/z are given in the titles

RYGB also induced more profound urinary bile acid changes compared to LGB and SG. An OPLS-DA model based on the bile acid profiles showed statistically significant differences between pre-op and post-op of RYGB in cohort 1 (Figure S4). The main differences were driven by an isomer of glucuronide-conjugated cholic acid, and glycine-conjugated primary bile acids (e.g. isomer of glycol-chenodeoxycholic acid and multiple isomers of glyco-cholic acids), which were present in higher levels at both SF and LF (Fig. 3). In contrast, some isomers of chenodeoxycholic acid sulphate and an isomer of glucuronide-chenodeoxycholic acid sulphate increased initially at SF but reduced to the pre-op level at LF. The same trend was also observed in glycine- and taurine-conjugated chenodeoxycholic acid sulphate. Whilst no robust model was obtained from cohort 2 RYGB patients, which was likely due to small group size, a similar trend was observed and the isomer of glucuronide-cholic acid, glycol-cholic acid, and tauro-chenodeoxycholic acid sulphate were found to be higher at 6-month post-RYGB compared to pre-op based on the univariate statistical analysis (Figure S4). Similarly, no robust OPLS-DA models were derived from SG and LGB groups (*p* > 0.05), although univariate statistical analysis showed increases in some bile acids following SG and LGB including an increase in an isomer of glycocholic acid for both procedures at LF and additional increases in serum tauro-cholic acid and in glyco- and tauro-chenodeoxycholic isomers at SF (Figure S5).

### RYGB induces dynamic metabolic changes in serum profiles over the first 12 months following the surgery

**Fig. 4:**
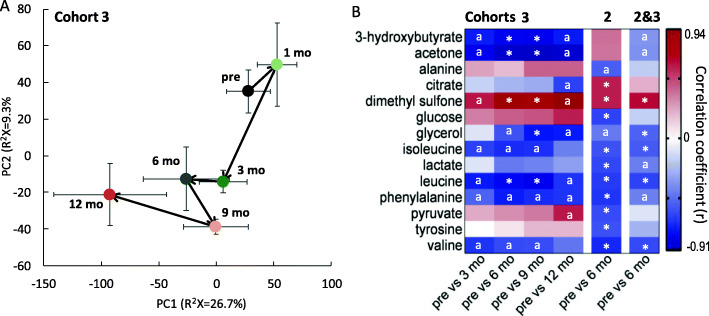
Trajectory PCA scores plot of serum ^1^H NMR spectra of RYGB patients from cohort 3 at pre-op and 1, 3, 6, 9 and 12 months post-op (**A**). Data are presented in mean ± SEM. The metabolites that were significantly different between pre-op and each of the post-op time points are summarized in the heatmap (**B**). The last two columns show the metabolite changes between pre-op and 6 months post-op in cohort 2 alone and in combined analysis of cohorts 2 and 3. The correlation coefficient (*r*) was derived from OPLS-DA models (Figure S6) and a positive correlation indicates higher relative concentrations of the metabolites in post-op compared to pre-op. *q*, the corrected p values using Benjamini-Hochberg multiple test correction are shown as *0.005 < *q* < 0.05. ^a^*p* < 0.05 and *q* > 0.05

Metabolic profiling of serum samples from cohort 3 enabled characterization of the dynamic biochemical trajectory post RYGB surgery as depicted in the PCA trajectory plot (Fig. 4A) Sequential OPLS-DA models were built to compare each of the post-op time points with pre-op (Figure S6) and showed that this metabolic shift was driven by high ketone bodies (e.g. 3-hydroxybutyrate and acetone) at pre-op and 1-month post-op, and a reduction of branched chain amino acids (BCAAs, e.g. leucine, isoleucine and valine) at 3–12 months post-op compared to pre-op (Fig. 4B). Dimethyl sulfone (DMSO_2_) was significantly increased at multiple post-op time points, whereas pyruvate was significantly higher at 12 months post-op compared to pre-op. Most of these changes were also observed in cohort 2. However, citrate was found to be higher and pyruvate was lower at post-op compared to pre-op in addition for cohort 2 (Fig. 4B). When combining the samples from cohorts 2 and 3, the most striking changes were higher circulating levels of dimethyl sulfone and decreased BCAAs post-surgery.

### RYGB surgery resulted in an altered microbiome and associated metabolic shift in the gut bacterial community

**Fig. 5:**
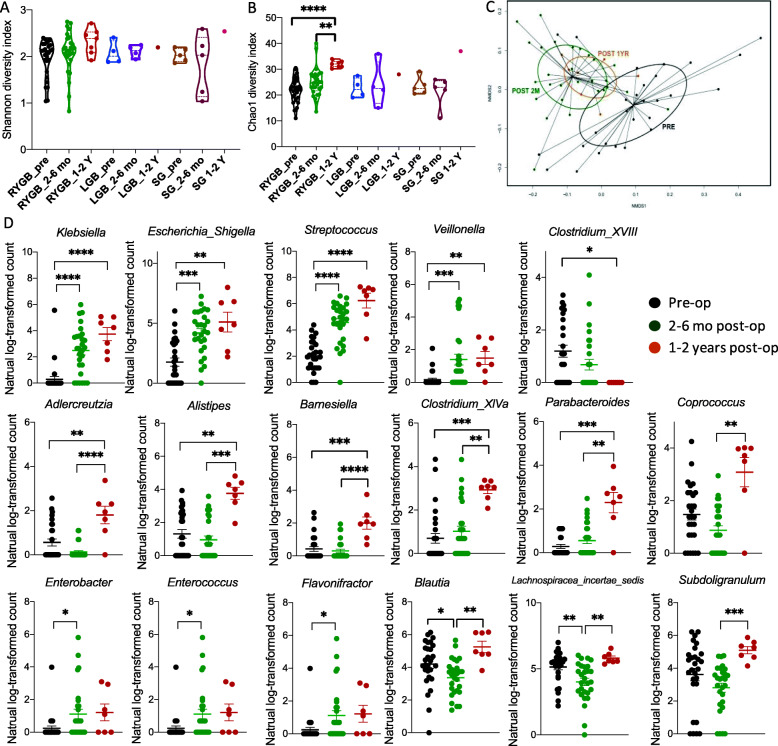
Shannon diversity index (**A**) and Chao1 diversity index (**B**) of faecal bacterial composition in different surgical groups at different time points (**A**, no statistical significance). An NMDS plot (**C**) of faecal bacterial composition of RYGB patients from cohort 1. PERMANOVA adjusted *p* = 0.0015 for pre-op vs. 2–6 months or 1–2 years post-op; *p* > 0.05 for 2–6 months vs. 1–2 years post-op. The natural log-transformed relative abundances of faecal bacteria from RYGB patients at pre-op (black), 2–6 months (green) and 1–2 years (orange) post-op (panel **D**). Data are presented in mean ± SEM. Kruskal-Wallis test was used for group comparisons and Dunn’s test was used for adjusting multiple comparisons. ****p < 0.0001, ***p < 0.001, **p < 0.01, *p < 0.05

Owing to the small group size of LGB and SG participants and the profound changes in host-bacterial crosstalk after RYGB, faecal bacterial and metabolic phenotyping analysis was carried out solely for the RYGB group. Although the alpha diversity, represented by Shannon diversity index, was not statistically different between different time points in RYGB patients (Fig. 5A), chao1 diversity index significantly increased at LF compared to pre-op and SF (Fig. 5B). RYGB surgery induced clear differences in the faecal microbiota based on the 16S rRNA gene sequencing analysis (Fig. 5C). A total of 17 bacterial genera belonging to 10 families were found to be significantly changed by RYGB surgery (Fig. 5C). The most striking alteration was that faecal bacterial genera *Klebsiella* and *Escherichia_Shigella* (*Enterobacteriaceae* family), *Streptococcus* (*Streptococcaceae* family) and *Veillonella* (*Veillonellaceae* family), all of which are small intestinal bacteria, were significantly increased at both short- and long-term follow-up. In contrast, genera, such as *Adlercreutzia* (*Coriobacteriaceae* family), *Alistipes* (*Rikenellaceae* family), *Barnesiella* and *Parabacteroides* (*Porphyromonadaceae* family) and *Clostridium_XIVa* and *Coprococcus* (*Lachnospiraceae* family) were significantly increased at long-term follow-up compared to pre-op and/or short-term post-op. *Enterobacter*, *Enterococcus* and *Flavonifractor* exhibited an initial increase and returned to the pre-op level at long-term follow-up, whereas *Blautia* and *Lachnospiracea_incertae_sedis* (*Lachnospiraceae* family), and *Subdoligranulum* (*Ruminococcaceae* family) showed an initial decrease before shifting back to the pre-op level. *Clostridium_XVIII* was reduced at the long-term but not short-term follow-up.

**Fig. 6:**
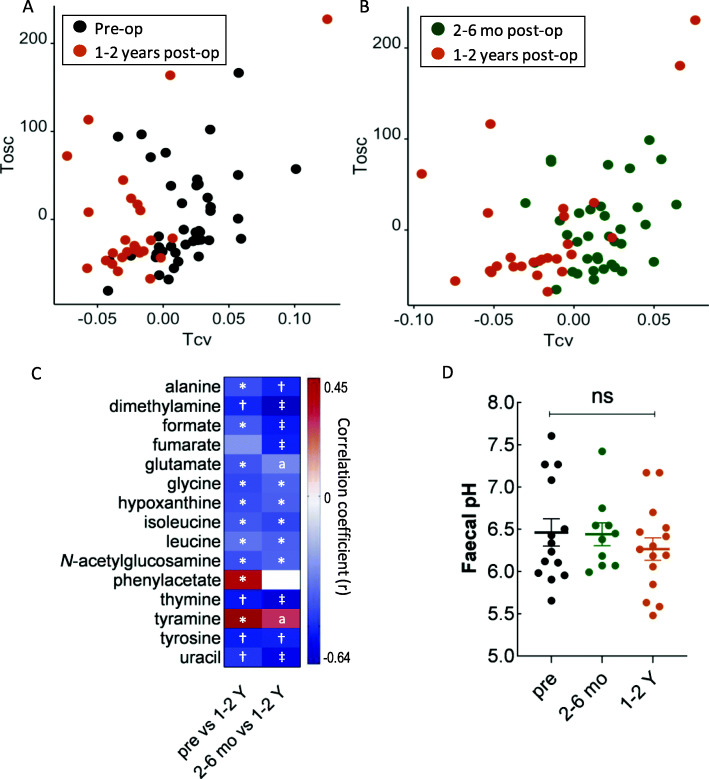
OPLS-DA cross-validated scores plots of faecal ^1^H NMR spectra of the RYGB patients from cohort 1 at pre-op, and 1–2 years post-op (**A**, Q^2^Y = 0.18; R^2^X = 31.8%, R^2^Y = 61.2%, *p* = 0.002), and at 2–6 months and 1–2 years post-op (**B**, Q^2^Y = 0.2; R^2^X = 30.7%, R^2^Y = 65.3%, *p* = 0.002). The metabolites that significantly contributed to the classification of different time points are shown in the heatmap (**C**). The correlation coefficient (*r*) was derived from OPLS-DA models and a positive correlation indicates higher relative concentrations of the metabolites in 1–2 years post-op compared to either pre-op or 2–6 months post-op. q, the corrected p values using Benjamini-Hochberg multiple test correction are shown as ‡*q* < 0.001, †*q* < 0.005, *q < 0.05. ^a^*p* < 0.05 and *q* > 0.05. Faecal pH of the RYGB patients at pre-op, 2-6 months and 1-2 Y post-op (**D**)

The faecal metabolome was also explored, and significant metabolic changes were found between pre-op and LF (Fig. 6A), and between SF and LF (Fig. 6B, ROC analysis of cross-validated scores is shown in Figure S1). The faecal concentrations of a range of amino acids (e.g. alanine, glutamate, glycine, isoleucine, leucine and tyrosine) were decreased post-surgery, whereas phenylacetate and tyramine, derived from phenylalanine and tyrosine, respectively, were found to be increased in the long-term follow-up. In addition, nucleobases, thymine and uracil were decreased, together with dimethylamine, formate, fumarate, hypoxanthine and *N*-acetylglucosamine at LF (Fig. 6C). RYGB was previously reported to reduce faecal pH in a mouse model and was suggested to be a factor in altering the gut microbiome structure post-surgery [8]; however, we did not observe any significant changes in faecal pH (Fig. 6D), suggesting that the pH is unlikely a key contributor to the compositional alteration of the bacterial community post RYGB in humans.

**Fig. 7:**
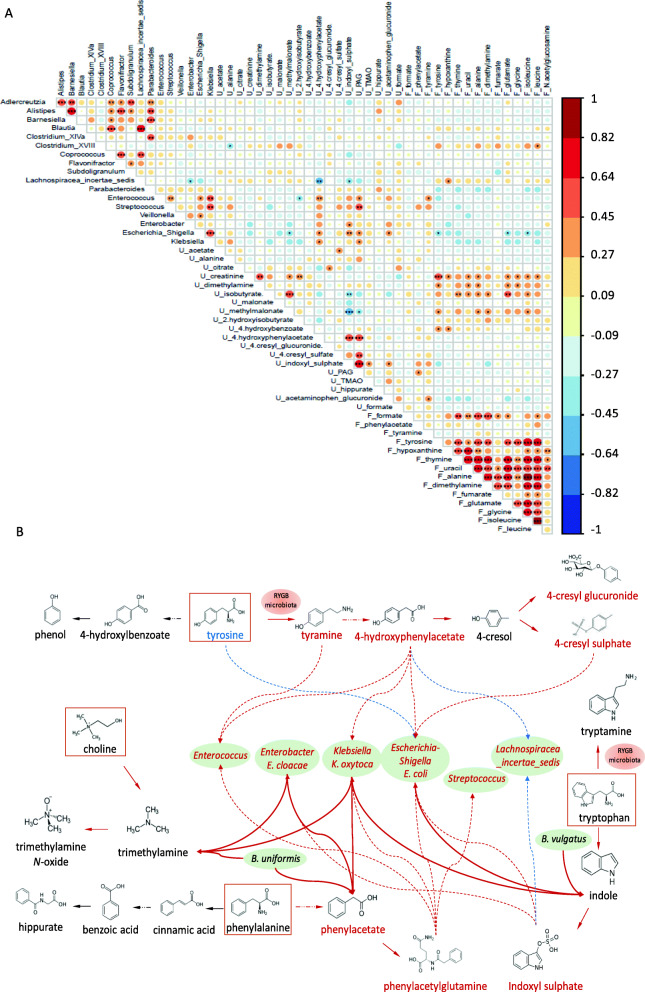
Spearman’s rank correlation of the significantly changed faecal bacterial abundances with faecal (F) and urinary (U) metabolites in RYGB patients from cohort 1 (**A**). The colour bar indicates the correlation coefficient *r* values and the p values post Benjamini-Hochberg multiple test correction are indicated as ***p < 0.001, **p < 0.01, *p < 0.05. Summary of metabolic pathways of tyrosine, phenylalanine, tryptophan and choline and associated gut bacteria (**B**). Bacteria and metabolite names depicted red indicate increased abundance or concentrations post-RYGB compared to pre-op. Bold solid red lines indicate metabolite production by the gut bacteria observed in the culture experiments. Red arrows between molecules indicate enhanced metabolic pathways following the RYGB surgery. The dashed line between the metabolites and gut bacteria indicates significantly positive (red) and negative (blue) correlation. The uneven dashed line indicates multiple reaction steps. The red ovals (RYGB microbiota) indicate metabolic transformation steps in the batch culture of faecal samples from RYGB donors

In addition to compositional analysis of the gut bacteria, we also carried out statistical correlation between the multi-compartment (urine and faeces) metabolic data and the taxonomic data to investigate the metabolic activities that were statistically associated with the compositional changes. We observed that most of the faecal amino acids were highly correlated with the faecal nucleobases, and metabolites involved in tyrosine, phenylalanine and tryptophan metabolism were highly correlated with each other (Fig. 7A). In addition, faecal tyrosine was significantly and positively correlated with urinary 4-hydroxybenzoate, creatinine, dimethylamine and methylmalonate, but negatively correlated with *Enterobacteriaceae* (e.g. *Escherichia_Shigella*). In contrast, tyramine, a decarboxylation product of tyrosine, was significantly and positively correlated with bacterial genera of *Enterococcus*. The precursor of PAG, phenylacetate, derived from phenylalanine, was also positively correlated with *Streptococcus*. Furthermore, TMAO was found to be positively correlated with *Enterobacter*, *Escherichia_Shigella* and *Klebsiella*.

In order to establish the direct relationship between the altered faecal bacterial species and the altered metabolite levels arising from RYGB surgery, we further studied the metabolic activities of whole faecal samples from healthy (*n* = 2) and RYGB (*n* = 2) donors, as well as bacterial isolates using an anaerobic batch culture technique. The statistical correlation and batch culture experimental observations are summarized in Fig. 7B. The production of tyramine was observed in faecal samples from the RYGB donors when cultured in tyrosine-supplemented media, but not in healthy donors. In contrast, healthy donors showed higher butyrate production compared to RYGB donors (Figure S7). Tryptamine was noted in one of the RYGB donors when cultured with tryptophan-supplemented media (Figure S7). Twelve representative bacterial species in healthy individuals (*Bacteroides uniformis* and *Bacteroides vulgatus*), obese patients (*Streptococcus cristatus*, *Streptococcus gallolyticus*, *Ruminococcus torques* and *Dorea longicatena*) and RYGB patients/animal models (*Shigella sonnei*, *Klebsiella pneumoniae*, *Escherichia coli*, *Enterobacter hormaechei*, *Enterobacter cloacae* and *Klebsiella oxytoca*) were selected for batch culture. Out of the 12 bacterial species, that were cultured based on their prominence in the intestines of healthy, RYGB or individuals with obesity (see Supplementary Information), *K*. *oxytoca*, *E*. *coli* and *B*. *vulgatus* were found to produced indole from tryptophan and *B*. *uniformis*, *K*. *oxytoca* and *E*. *cloacae* produced phenylacetate and TMA from phenylalanine and choline, respectively (Figure S8). These results confirmed the statistical correlation and supported the observations of higher levels of urinary host-bacterial co-metabolites after RYGB surgery.

## Discussion

Our data shows that RYGB is more effective for inducing weight loss compared to LGB and SG, which is consistent with several randomized and prospective studies [12, 13]. Patients who underwent SG also showed a notable reduction in BMI; however, it did not reach statistical significance likely owing to the relatively small number of patients. RYGB surgery also induced the most profound metabolic changes compared to LGB and SG and some of these changes were statistically and biologically associated with bacterial genera *Klebsiella*, *Escherichia_Shigella*, *Streptococcus* and *Veillonella*. The relative abundances of these bacteria were elevated following RYGB, which are in an agreement with previously published studies [5, 6]. *Klebsiella* and *Escherichia_Shigella* are Gram-negative and facultative anaerobic bacteria belonging to *Enterobacteriaceae* family, which is present at a very low abundance (<<10^8^ CFU/g) in a healthy colon but has been consistently reported to increase in patients with inflammatory bowel disease and colorectal cancer (CRC) [14, 15]. In addition, *Streptococcus*, Gram-positive bacteria from *Bacilli* class, and *Veillonella*, Gram-negative anaerobic bacteria, were also found to be associated with CRC (*Streptococcus tigurinus* and *Streptococcus dysgalactiae*) [15]. Epidemiological research demonstrated that bariatric surgery was associated with increased CRC risk [16, 17], which could be contributed by the high abundances of these bacteria owing to their pro-inflammatory effects [18]. *Klebsiella* spp. isolated from the saliva have been shown to induce T helper 1 cell proliferation when colonizing in the gut, demonstrating that colonization of the oral bacteria in the gut could result in the intestinal inflammation [18]. The driving factors of increased Gammaproteobacteria post RYGB remain inconclusive. However, excessive transit of swallowed O_2_ in the colon (so called air hypothesis [19]), altered gastric and intestinal emptying, increased gastric pH due to fewer hydrochloric acid pumps in the gastric pouch [20], and disturbed nutrient exposure in the gut could contribute to this bacterial shift. Furthermore, the undigested food flow post RYGB can trigger intestinal hyperosmolarity [21], which has been shown to prevent commensal bacterial growth and increase the growth of Gammaproteobacteria [22]. The observation that faecal cultures from healthy donors produced higher concentrations of butyrate than the post-RYGB donors also suggests that the shift in microbiome composition may represent an adverse health effect since colonic butyrate has been inversely associated with CRC [23]. Our findings highlight a critical need for understanding the consequences of altered host and microbial metabolism for patients undergoing RYGB surgery to prevent long-term health risks.

Urinary concentrations of TMAO at the SF of RYGB increased in both UK and US cohorts but normalized at LF. TMAO is an oxidative product of TMA, primarily catalysed by hepatic flavin-containing monooxygenase isoform 3 (FMO3) [24].TMA is mainly produced from dietary choline and carnitine by the gut bacterial enzymes, choline TMA lyase and carnitine oxidoreductase, respectively and can be degraded to dimethylamine in the gut, underscoring the impact of RYGB surgery on enhanced bacterial degradation of choline [24]. More than 2-fold increase in plasma concentrations of TMAO following RYGB or duodenal switch have previously been reported without increased dietary intake of choline and carnitine [25], which is consistent with our observation since TMAO is commonly excreted unchanged in urine within 24 h. Many bacteria from *Enterobacteriaceae* have capacity to produce TMA [26] and we demonstrated via in vitro culture experiments that *K*. *oxytoca* and *E*. *cloacae* from this bacterial family were able to produce TMA from choline. TMAO is associated with vascular aging, atherosclerosis, cardiovascular and neurological disorders [24, 27]; however, bariatric surgery significantly reduces the cardiovascular risk through a proposed entero-cardiac axis, including modulation of gut hormones and other intermediaries acting on the vascular system and directly on the cardiac myocardium [28], and therefore, this association warrants future studies. Furthermore, TMAO has been associated with inflammation, manifested by increased oxidative stress and pro-inflammatory cytokines (e.g. IL-6 and TNF-α), which could have a long-term impact on the patients following RYGB. In addition, a link between choline metabolism and CRC was characterized by an overabundance of choline TMA lyase gene in CRC patients [15], suggesting that elevated choline metabolism following RYGB could be associated with higher CRC risk. Moreover, our faecal batch culture experiments showed a lower ability of bacterial butyrate production in the RYGB patients compared to healthy donors, but this trend was not as evident in the propionate production. Butyrate, a bacterial (primarily *Clostridium clusters IV* and *XlVa*) fermentation product from dietary fibre and amino acids (e.g. glutamate and lysine via *Fusobacterium* spp. based on an *in silico* analysis) [29], provides primary energy source of the colonic epithelial cells and inhibits colonic neoplasia and inflammation, and it is believe to reduce CRC risk [30]. It is noted that while *Clostridium XIVa* increased at long-term follow-up, faecal butyrate levels did not differ between pre- and post-op time points, which could be due to insufficient dietary fibre intake in the bariatric patients. The observed higher choline metabolism and reduced butyrate production capacity may partially explain the previously reported increased CRC risk in RYGB patients. However, TMA is a known uremic toxin and has been reported to decrease the viability of human vascular smooth muscle cells at concentrations of 500 μmol/L, whereas TMAO had no effect at 500 times this concentration [31]. Both methylamine and trimethylamine were correlated with toxicity in mouse lymphoma cell lines when incubated with faecal slurries from rats post-RYGB surgery, again highlighting the potential role of enhanced microbial TMA production in contributing to a cytotoxic environment [32]. *Veillonella* was significantly increased post-op, which could contribute to the propionate production from lactate in the faecal batch culture [33]. Propionate has been shown to induce secretion of satiety gut hormones, e.g. GLP-1 and PYY, which are elevated post RYGB surgery [34].

Bacterial metabolism of aromatic amino acids was elevated following RYGB surgery and batch culturing RYGB microbiota demonstrated conversion of tyrosine to tyramine, and tryptophan to tryptamine, suggesting that tyrosine decarboxylase and l-tryptophan decarboxylase activities were enhanced and resulted in higher faecal concentrations of the biogenic amines following RYGB. Tyramine is one of the most abundant biogenic amines in fermented food and beverages and showed the genotoxicity to intestinal cells [35]. Tyramine can be metabolized into 4-hydroxyphenylacetate and then 4-cresol by several enzymes including 4-hydroxyphenylpyruvate decarboxylase and 4-hydroxyphenylacetate decarboxylase [36], which supports our findings of increased urinary 4-hydroxyphenylacetate post-RYGB and suggests enhanced activities of a range of decarboxylases. 4-Cresol is subsequently conjugated with sulphate or glucuronide in the liver before excreted in the urine, which is consistent with elevated levels of urinary 4-cresyl sulphate and 4-cresyl glucuronide following RYGB. In contrast, the pathway of tyrosine to phenol did not appear to be favoured, evidenced by decreased urinary concentrations of 4-hydroxybenzoate at LF. Smith and Macfarlane reported that phenol production reduced, whereas 4-cresol increased when culturing human faecal bacteria with peptides compared to free amino acids with presence of starch [37]. Together with our observations, we speculate that incomplete digestion of proteins in the foregut may increase the bioavailability of peptides, but less likely free amino acids, in the distal colon, resulting in higher protein putrefaction and production of 4-hydroxylphenylacetate and 4-cresol.

Another finding of interest was increased concentrations of phenylacetate in faeces and its glutamine-conjugated compound, PAG, in urine following RYGB. Whilst phenylacetate was strongly correlated with *Streptococcus*, we showed empirically that *B*. *uniformis*, *K*. *oxytoca* and *E*. *cloacae* metabolized phenylalanine into phenylacetate, and the relative abundance of the genera containing the latter two bacterial species increased post-RYGB. In contrast to PAG, an increased trend of urinary hippurate was observed post-RYGB, which is in agreement with inversed association of hippurate and PAG with BMI observed in an epidemiology study [38]. Hippurate is mainly formed by hepatic conjugation of glycine and benzoic acid; benzoic acid is derived from phenylalanine or obtained directly from the diet [39]. A significant increase in PAG and a moderate increase in hippurate indicated that bacterial phenylalanine metabolism may have shifted towards phenylacetate production following RYGB.

Further evidence of modulation of the gut microbiome after RYGB surgery was the observation of increased urinary concentrations of indoxyl sulphate, the precursor of which is indole, derived from bacterial metabolism of tryptophan [39]. Indoxyl sulphate was found to be positively correlated with *Enterococcus*, *Streptococcus*, *Escherichia_Shigella* and *Klebsiella*. The in vitro culture experiment further confirmed the production of indole from tryptophan by *K*. *oxytoca*, *E*. *coli* and *B*. *vulgatus*, consistent with the increased abundance of *Klebsiella* and *Escherichia_Shigella* and their consequential contribution to the production of indoxyl sulphate. Indoxyl sulphate, together with 4-cresyl sulphate, 4-cresyl glucuronide and TMAO have been classified as uremic toxins [40]. Whilst TMAO is quickly cleared via tubular secretion, the clearance of the other 3 compounds, which bind with proteins, mainly albumin, is slower [41]. Incubation of mouse lymphoma cell lines with 4-cresyl glucuronide was anticorrelated with cell growth [32], again supporting the hypothesis that post-surgical alterations in the microbiome and its metabolites contributes to a cytotoxic environment within the gut. The gut bacterial contribution to increased levels of these compounds may exert long-term health risk for RYGB patients. However, on the other hand, vitamin K, produced by *Veillonella*, which increased post-RYGB, has a nephroprotective role [40].

To our best knowledge, it is the first time that elevated urinary concentrations of hydroxymyristic acid, 3-hydroxydodecanedioic acid and 3-hydroxytetradecanedioic acid have been observed in the RYGB-operated patients, indicating a disturbance in mitochondrial β-oxidation of medium- and long-chain fatty acids. Seridi et al. reported a decrease in plasma myristic acid following RYGB procedure [42]. Urinary 3-hydroxy dicarboxylic acids have been found in patients with ketoacidosis, suggesting an incomplete β-oxidation [43]. Excessive urinary excretion of 3-hydroxytetradecanedioic acid was also reported in a child with a genetic defect in mitochondrial enzyme, long-chain 3-hydroxyacyl-coenzyme A (CoA) dehydrogenase (LCHAD) [44], in children with persistent wheeze/asthma [45] and in patients treated with the drug hopantenate, where 3-hydroxytetradecanedioic acid was indicative of an acute intoxication response associated with the treatment during prolonged fasting or malnutrition [46]. Another study showed a lower intracellular concentration of the metabolite and a potentially higher release from the human L02 liver cells as a toxic response to pekinenal from the herb *Euphorbia pekinensis* Rupr [47]. The increased excretion of these medium and long-chain 3-hydroxy-dicarboxylic acids could be owing to the inhibition of LCHAD and median- and short-chain 3-hydroxyacyl-coenzyme A (CoA) dehydrogenases (SCHAD), which use NAD^+^ as a cofactor to catalyse the oxidation of l-3-hydroxyacyl-CoA to 3-ketoacyl-CoA [48]. Mice without SCHAD (*hadh*^−/−^) exhibit lower body weight and fat mass compared to wildtype when fed a high-fat diet, and the pancreatic islets of *hadh*^−/−^ mice showed a higher insulin secretion in response to glucose [48]. Therefore, the inhibition of SCHAD could be associated with weight loss post RYGB surgery. Furthermore, the association between the foetal genetic deficiency of LCHAD and pregnancy-specific disorders, such as acute fatty liver and preeclampsia, has been reported [49], and this may also therefore represent one additional mechanism in reducing the burden of non-alcoholic fatty liver disease after RYGB. Whilst the urinary phenotype of the babies, born from mothers who previously had RYGB surgery, has been shown to be similar to their mothers’ host-bacterial co-metabolite signatures [5], very little is known regarding the impact of the disturbed fatty acid metabolism in maternal bariatric surgery patients on their offspring.

Higher serum levels of ketone bodies were observed at pre-op and 1-month post-op compared to the other post-op time points in cohort 3. This increase could be due to the low-calorie diet the patients had prior to the surgery and low-calorie intake at the initial stage following the surgery. Consistent with previous findings [5], serum BCAA concentrations, which are known to increase insulin resistance, were reduced in both cohorts. Another significant change in serum was increased DMSO_2_ concentrations (1.7-fold in cohort 2 and 3.0-fold in cohort 3 at 6 months) following RYGB, which was consistent with a previous publication where the increase was the most prominent in distal RYGB compared to proximal RYGB and LSG [50]. DMSO_2_ can be derived from diet (e.g. rye, onions and asparagus), specifically host metabolism and host-bacterial metabolism of methionine [51]. However, the consistent reports on increased serum levels of DMSO_2_ across different RYGB patient cohorts have led us to speculate that this change is associated with the gut bacterial disturbance we observed. Like the aromatic amino acids (*vide supra*), dietary methionine could escape the small intestinal absorption due to anatomic changes of RYGB surgery and undergo catabolism by the colonic bacteria. It is estimated that 20% of dietary methionine is metabolized in an anatomically normal gut [51]. Bacteria from *Enterobacteriaceae* family, which increases post RYGB, were found to contain *megL* gene, which encodes l-methionine gamma-lyase [52]. This enzyme catalyses the conversion of methionine to methanethiol, which is subsequently metabolized into DMSO_2_ by the host [51]. Therefore, the increased concentrations of DMSO_2_ in serum is highly likely to result from a host-bacterial crosstalk following RYGB. Whilst the biological function of DMSO_2_ is yet fully understood, its use for treating snoring has been proposed [53]. RYGB surgery is the most effective treatment for obstructive sleep apnoea (OSA) [54] and it is possible that DMSO_2_ plays a role in improving the symptom of OSA.

Integration of metabolic phenotypic and microbiomic data were used to investigate the impact of RYGB surgery in three independent cohorts of patients. The metabolic consequences of the surgically induced alterations in the microbiome were further pursued using a suite of in-vitro culture techniques. This involved the incubation of key bacterial cultures, or faecal slurries from control and RYGB-operated individuals with metabolic substrates in order to establish the ability of selected bacteria or microbiomes to synthesize the metabolites that characterize the post-RYGB phenotype. Whilst the number of participants enrolled was larger than for most published studies to date, the numbers recruited for cohorts 2 and 3 were small and increased numbers would have served to strengthen the characterization of metabolic changes in the serum and to evaluate regional differences induced by bariatric surgery. Moreover, incubation of faecal slurries from a greater number of RYGB patients and weight-matched control participants would have added depth to the study and the variation between RYGB patients requires further investigation. Approximately 20% of patients undergoing bariatric surgery are male, and male patients show significantly lower weight loss and rates of comorbidity resolution compared to female [55]. Therefore, further studies with a larger recruitment of male patients should be carried out to systematically explore sex-differences in the metabolic and microbial responses induced by bariatric surgery. Nevertheless, the combination of metabolic phenotyping with substrate profiling of the in vitro experiments strongly corroborate the role of the altered microbiome in driving metabolic changes post-surgery.

## Conclusion

In summary, we demonstrated that RYGB induces a matrix of effects, including enhanced amino acid metabolism of tyrosine, phenylalanine, tryptophan and methionine, and altered mitochondrial fatty acid metabolism. This metabolic activity likely contributes to the many downstream physiological changes after bariatric surgery beyond that of ‘simply weight loss’ or only ‘anti-diabetic’ effects in multiple organ systems beyond the gastro-intestinal system (such as the liver, kidney, heart and the brain). We showed that the altered gut microbiome contributes to the host-bacterial co-metabolite signatures in urine and faeces following RYGB surgery and that these changes are likely driven by a subset of bacterial species that are favoured by the post-RYGB gut environment. The impact of RYGB-induced metabolic and bacterial changes on the long-term health of the patients warrants further studies to evaluate the patient cohorts who would derive most clinical and physiological benefits from this procedure. Better understanding the profound host-bacterial cross talk after bariatric operations could therefore allow the better selection of patients for current procedures and lead to the genesis of the next generation of weight loss and metabolic protecting and enhancing therapies.

## Supplementary Information

**Additional file 1: Figure S1.** ROC analysis of cross-validated scores from OPLS-DA models. (A) NMR analysis of urine; Tcv1 and Tcv2 represent the first and second PLS component when 2 PLS components were applied to separate 3 classes; (B) LC-MS analysis of urine; (C) bile acid analysis of urine; (D) NMR analysis of serum; (E) NMR analysis of feces. **Figure S2.** Relative metabolite levels in urine from cohorts 1 (A) and 2 (B), plasma from cohorts 3 (C) and 2 (D), and feces from cohort 1 (E). Metabolite levels are indicated by relative peak heights from the median fold normalized spectra of urine and feces and non-normalized serum spectra. Error bars are presented in SEM. a.u. stands for arbitrary unit. **Figure S3.** OPLS-DA cross-validated scores plots of urinary ^1^H NMR spectra of the RYGB and LGB patients from cohort 3 at pre-op (Q^2^Y=0.49; R^2^X=12.35%; R^2^Y=89.6%; *p* = 0.006), and 6-month post-op (Q^2^Y=0.44; R^2^X=30.66%; R^2^Y=95.2%; *p* = 0.038). The metabolites that significantly contributed to the classification of different time points are shown in Fig. 2C. **Figure S4.** OPLS-DA cross-validated scores plots of urinary reverse-phase liquid chromatography-mass spectrometry profiles of the bariatric patients from cohort 1 in ESI positive mode (Q^2^Y=0.46; R^2^X=24.8%; R^2^Y=73.4%; CVANOVA p = 2.9 x 10^-14^) and ESI negative mode (Q^2^Y=0.52; R^2^X=14%; R^2^Y=76.6%; CVANOVA p = 6.2 x 10^-17^). The identified metabolites that significantly contributed to the classification between pre-op (black) and 2-6 months post-op (green) are listed in the table. Positive correlation (r) represents higher relative concentrations of these metabolites at the post-op compared to pre-op. p[1] is loadings from the OPLS-DA models. Coefficient of variation (CV) was calculated based on the quality control (QC) samples to evaluate the analytical variation of the features. **Figure S5.** OPLS-DA cross-validated scores plots of urinary bile acid profiles of the RYGB patients from cohort 1 at pre-op (black), 2-6 months (green) and 1-2 years (orange) post-op (A. Q^2^Y=0.21; R^2^X=16.9%; R^2^Y=48.9%; CVANOVA *p* = 5.8x10^-9^). Bar plots of relative intensities of urinary bile acids identified from cohort 2 RYGB patients at pre-op (black) and 6 months post-op (green). Wilcoxon matched pairs signed rank test was used. ** 0.001< *p* <0.01. Data were presented as mean±SEM. The bile acids and their retention time (min) and m/z were given in the titles. **Figure S6.** Relative intensities of urinary bile acids from sleeve gastrectomy (SG) and laparoscopic gastric banding (LGB) patients from cohort 1 at pre-op (black), 2-6 months (green) and 1-2 years post-op (orange). Kruskal-Wallis test was used, and Dunn’s test was used for adjusting multiple comparisons. The adjusted *p* values: ****, *p* < 0.0001, ***, *p* < 0.001, ** *p* < 0.01, * *p* < 0.05. Data were presented as mean±SEM. The bile acids and their retention time (min) and m/z were given in the titles. **Figure S7.** OPLS-DA cross-validated scores plots of serum ^1^H NMR CPMG spectra of the RYGB patients from cohort 3 between pre-op and 3 (A, Q^2^Y=0.69; R^2^X=35.1%; R^2^Y=98.2% *p* = 0.002), 6 (B, Q^2^Y=0.63; R^2^X=40.4%; R^2^Y=97.9% *p* = 0.002), 9 (C, Q^2^Y=0.80; R^2^X=37.9%; R^2^Y=98.6% *p* = 0.002) or 12-month (D, Q^2^Y=0.68; R^2^X=45.5%; R^2^Y=98.3% *p* = 0.008) post-op. OPLS-DA cross-validated scores plots of serum ^1^H NMR CPMG spectra of the RYGB patients between pre-op and 6-month post-op of cohort 2 (E, Q^2^Y=0.56; R^2^X=37.7%; R^2^Y=87.7% *p* = 0.002) or combined cohorts 2 and 3 (F, Q^2^Y=0.25; R^2^X=31.4%; R^2^Y=72.9% *p* = 0.002). **Figure S8.** Partial ^1^H NMR spectra of faecal batch culture media supplemented with tyrosine or tryptophan at 0 (black), 7 (blue), 24 (red) and 48 (pink) hours. Triplicates were carried out and the median spectra at each time point are shown. The top and middle panels are from healthy and RYGB donors, respectively. The bottom panel is from one of the RYGB donors. X axis is chemical shift in ppm and Y axis is peak intensities. **Figure S9.** Partial ^1^H NMR spectra of bacterial isolate culture in a defined simple media supplemented with phenylalanine (top panel), tryptophan (middle panel) or choline (bottom panel). Triplicates were carried out and all media spectra are shown. Black: control media; blue: *B. uniformis*; red: *K. oxytoca,* pink: *E. cloacae; green: E. coli*; light blue: *B. vulgatus.* X axis is chemical shift in ppm and Y axis is peak intensities. **Table S1.** Summary of exemplar human clinical studies investigating the metabolic effects of bariatric surgery to date. **Table S2.** Summary of the number of patients and body mass index (BMI) at each time based on types of bariatric surgery. Values are shown as mean±SD. Kruskal-Wallis ANOVA test was used to test the significance of BMI changes across the time points in cohorts 1 and 3, while Friedman test was used for cohort 2 for paired analysis.

## Data Availability

Data will be made available once the manuscript is accepted.
