# Supplementary material for: Roux-en-Y gastric bypass-induced bacterial perturbation contributes to altered host-bacterial co-metabolic phenotype
Source: Microbiome. 2021 Jun 14;9:139. doi: 10.1186/s40168-021-01086-x (PMC8201742; doi:10.1186/s40168-021-01086-x)
Supplement: Supplementary file 2 — Additional file 1: Figure S1. ROC analysis of cross-validated scores from OPLS-DA models. (A) NMR analysis of urine; Tcv1 and Tcv2 represent the first and second PLS component when 2 PLS components were applied to separate 3 classes; (B) LC-MS analysis of urine; (C) bile acid analysis of urine; (D) NMR analysis of serum; (E) NMR analysis of feces. Figure S2. Relative metabolite levels in urine from cohorts 1 (A) and 2 (B), plasma from cohorts 3 (C) and 2 (D), and feces from cohort 1 (E). Metabolite levels are indicated by relative peak heights from the median fold normalized spectra of urine and feces and non-normalized serum spectra. Error bars are presented in SEM. a.u. stands for arbitrary unit. Figure S3. OPLS-DA cross-validated scores plots of urinary 1H NMR spectra of the RYGB and LGB patients from cohort 3 at pre-op (Q2Y=0.49; R2X=12.35%; R2Y=89.6%; p = 0.006), and 6-month post-op (Q2Y=0.44; R2X=30.66%; R2Y=95.2%; p = 0.038). The metabolites that significantly contributed to the classification of different time points are shown in Fig. 2C. Figure S4. OPLS-DA cross-validated scores plots of urinary reverse-phase liquid chromatography-mass spectrometry profiles of the bariatric patients from cohort 1 in ESI positive mode (Q2Y=0.46; R2X=24.8%; R2Y=73.4%; CVANOVA p = 2.9 x 10^-14^) and ESI negative mode (Q2Y=0.52; R2X=14%; R2Y=76.6%; CVANOVA p = 6.2 x 10^-17^). The identified metabolites that significantly contributed to the classification between pre-op (black) and 2-6 months post-op (green) are listed in the table. Positive correlation (r) represents higher relative concentrations of these metabolites at the post-op compared to pre-op. p[1] is loadings from the OPLS-DA models. Coefficient of variation (CV) was calculated based on the quality control (QC) samples to evaluate the analytical variation of the features. Figure S5. OPLS-DA cross-validated scores plots of urinary bile acid profiles of the RYGB patients from cohort 1 at pre-op (black), 2-6 months (green) a [file 40168_2021_1086_MOESM2_ESM.docx]

**Supplementary information**

**Roux-en-Y Gastric bypass-induced bacterial perturbation contributes to altered host-bacterial co-metabolic phenotype**

Jia V Li^1^, Hutan Ashrafian^2^, Magali Sarafian^1^, Daniel Homola^1^, Laura Rushton^1,3^, Grace Barker^1^, Paula Momo Cabrera^1^, Matthew R Lewis^4^, Ara Darzi^2^, Edward Lin^5^, Nana Adwoa Gletsu-Miller^6^, Stephen L Atkin^7^, Thozhukat Sathyapalan^8^, Nigel J Gooderham^1^, Jeremy K Nicholson^9^, Julian R Marchesi^1,3^, Thanos Athanasiou^2^, Elaine Holmes^1,9^

^1^Division of Digestive Disease, Department of Metabolism, Digestion and Reproduction, Imperial College, London SW7 2AZ, UK

^2^Division of Surgery, Department of Surgery and Cancer, Imperial College London, London SW7 2AZ, UK

^3^School of Biosciences, Museum Avenue, Cardiff University, Cardiff, CF10 3AT, UK

^4^Division of Systems Medicine, Department of Metabolism, Digestion and Reproduction, Imperial College, London SW7 2AZ, UK

^5^Division of General and Gastrointestinal Surgery, Department of Surgery, Emory University School of Medicine, Atlanta, Georgia 30322, USA

^6^Department of Applied Health Science, School of Public Health, Indiana University Bloomington, 1025 E 7th Street, Bloomington, IN 47405, USA

^7^RCSI Bahrain, Adiya, Kingdom of Bahrain

^8^Department of Academic Endocrinology, Diabetes and Metabolism, Hull-York Medical School, Hull, UK

^9^Centre for Computational and Systems Medicine, The Health Futures Institute, Murdoch University, Harry Perkins Building, Perth, WA6150, Australia

**Correspondence:**

Elaine Holmes, [elaine.holmes@imperial.ac.uk](mailto:elaine.holmes@imperial.ac.uk)

**Methods**

**Sample preparation, NMR spectroscopic analysis of urine, feces and serum, and spectral data analysis**

Urinary samples were defrosted at room temperature for 4 hours and vortexed thoroughly. One millilitre of urine was aliquoted into a 1.5 ml microcentrifuge tube and centrifuged at 13,000 *g* at 4 °C for 10 min. A total of 540 µl was mixed with 60 µl of 1.5 M potassium phosphate buffer in D_2_O (pH = 7.4, 0.1% 3-(trimethylsilyl)-[2,2,3,3-^2^H_4_] propionic acid sodium salt (TSP) and 2 mM sodium azide) and transferred into an NMR tube with an outer diameter of 5 mm. ^1^H NMR spectra were acquired using a Bruker Avance III console combined with a 14.1 T magnet for ^1^H 600 MHz (Bruker Biospin, Germany) at a temperature of 300 K. A standard NMR pulse sequence (recycle delay-90°-t_1_-90°-t_m_-90°-acquisition) was used to acquire standard one-dimensional ^1^H NMR spectral. The water peak suppression was achieved using selective irradiation during a recycle delay of 4 s and t_m_. A 90° pulse was optimized for each sample to around 10 μs. A total of 32 scans were collected into 64 k data points with a spectral width of 20 p.p.m. ^1^

Approximately 300 mg of fecal samples were weighted and extracted using 3 mL of a mixture of water: methanol: chloroform (1:1:1). Following a thorough vortex, samples were spun at 2000 *g* at 4°C for 10 min. The upper layer (aqueous phase) and the lower layer (organic phase) were transferred separately into glass tubes. The undissolved residuals were extracted again with 3 ml of the mixture using the same procedures described above. The aqueous phase extracts were dried in a speed vacuum centrifuge for 8 hours at 30°C and stored at -40°C. Prior to NMR spectroscopic analysis, the dry extracts were suspended in 500 µL H_2_O (HPLC grade) and 400 µL was taken to mix with 250 µL 0.2 M sodium phosphate buffer (100% D_2_O, 1 mM TSP, 3 mM NaN_3_, pH = 7.4). A total of 600 µL of the supernatant was transferred to an NMR tube with an outer diameter of 5 mm following 10 min centrifugation at 18,000 *g* at 4°C. 600 MHz Avance DRX NMR (Bruker Biospin, Germany) was used and the same standard NMR pulse sequence used for urinary spectra acquisition was used for fecal water extracts, with a recycle delay of 2 s, t_1_ of 3 µs and t_m_ of 100 ms. A 90° pulse was optimized for each sample to around 10 μs. A total of 256 scans were collected into 64 k data points with a spectral width of 20 p.p.m.^2^

Serum samples were thaw defrosted at room temperature for 1 hour and vortex thoroughly, followed by centrifugation at 18,000 *g* at 4°C for 10 min. A total of 300 mL of supernatant was mixed with 300 µL of sodium phosphate buffer. The NMR spectroscopic analysis was carried out at 310 K and the experimental parameters were described previously.^1^

The spectral data were automatically phased, baseline-corrected and calibrated to TSP δ^1^H 0 before being imported to MATLAB R2014a with a resolution of 0.0005 p.p.m. for pre-processing and multivariate statistical analysis. The spectral regions containing noise, water peak or urea signal, e.g. 0-0.3 ppm, 4.7-4.88 ppm and 5.45-6.32 ppm in urinary spectra, 0-0.25 ppm and 4.72-4.9 ppm in fecal spectra, and 0-0.5 ppm and 4.52-5.0 ppm in serum spectra were removed. Urinary spectra were subsequently aligned using recursive segment-wise peak alignment method.^3^ The urinary and fecal spectral data were normalized using median fold normalization method, whereas serum spectra were unnormalized. The principal component analysis (PCA) and orthogonal projections to latent structures-discriminant analysis (OPLS-DA) were carried out based on mean centered and unit variance scaled datasets.

**Sample preparation, UPLC-MS-based global metabolic profiling of urine and data analysis**

A total of 114 urinary samples from 57 patients at pre and short-term follow-up after bariatric surgery were analyzed using ultra performance liquid chromatography-Mass Spectrometry. A quality control (QC) sample, pooled from a small portion of the study samples, was prepared for column conditioning and quality control as described by Want et. al.^4^ All samples including QC were diluted with water 1:1 (v:v) and centrifuged at 18,000 *g*, 4°C for 10 mins. The supernatant was transferred to glass vials for UPLC-MS analysis.

Profiling was conducted using a reversed phase (RP) LC method described previously^4^, with an Acquity UPLC coupled to a Q-ToF Premier (Micromass/Waters Corp., Manchester UK) and electrospray interface operating in either the positive or negative ionization mode. Dynamic range extension was utilized, expanding the linear range of the detector by collecting and combining both full strength and attenuated strength signals (a correction factor was automatically applied by the instrument software to the latter) to allow for accurate intensity measurements of otherwise saturated peaks. Data was collected across the 40 to 1000 m/z range with a scan rate of 0.2 s/scan and a minimum (0.02 s) inter-scan delay. The electrospray ionization (ESI) source capillary and sampling cone voltages were 3000 and 30 volts, respectively for positive mode ionization and 2500 and 25 volts, respectively for negative mode ionization. A source temperature of 120 °C was used, along with a desolvation flow of nitrogen gas at 800 L/h and 400 °C. Samples were analyzed in a randomized order and the QC sample was injected once every 10 injections of the study samples.

Data extraction *via* feature detection, alignment, grouping, and integration was performed on each dataset (positive and negative ionization mode) using XCMS. The processing variables used for centWave feature detection were peak width of 2-10 sec and signal to noise ratio of ≥10. The grouping function was performed using the nearest method with retention time and mass axis grouping boundaries of 12 seconds and 0.07 Da., respectively. Median fold change normalization was applied to the filtered dataset.^5^ The resulting matrix of feature intensities across all samples and feature groups was imported to SIMCA-P+ 15 for multivariate statistical analysis. UV scaling was applied to the data prior to the modelling.

**Sample preparation, MS-based bile acid profiling of urine, and data analysis**

Urinary samples were thawed at 4°C overnight and centrifuged at 18,000 *g* for 15 min at 4°C. A total of 100 µL of the supernatant from each sample was transferred 0.5 mL Eppendorf 96-deepwell plates and 300 µL of ice-cold methanol was added to each well for protein precipitation. All plates were heat-sealed (Thermo Fisher Scientific, Hertfordshire UK), vortexed for 30 min at 4°C using an Eppendorf MixMate at 1400 rpm and incubated for 20 min at -20°C, followed by centrifuging at 3486 *g* for 15 min at 4°C. A total of 200 µL of supernatant was transferred to Eppendorf 350 µL microplates, which were subsequently heat-sealed with thermofoil prior to analysis. Another 20 µL of supernatant from each urinary sample was pooled together in glass beakers to form a QC sample (total volume=1420 µL for each type of biofluid). A total of 4260 µL of ice-cold methanol was added to the glass beaker for protein precipitation according the aforementioned incubation steps. The supernatant from the QC sample was transferred into multiple wells in the Eppendorf 350 µL microplates.

Bile acid profiling was carried out using ACQUITY UltraPerformance Liquid Chromatography (UPLC) coupled with a Xevo G2-S Q-ToF mass spectrometer (Waters Ltd.). The injection volume of all samples was 10 μL. To minimize injector carry-over, 3 wash cycles of weak (H_2_O:2-propanol, 9:1, v:v) and strong (2-propanol) solvent preparations were performed simultaneously with sample analysis. ACQUITY BEH C8 column (1.7 μm, 100 mm × 2.1 mm) was used at an operating temperature of 60°C. The mobile phase A consisted of acetonitrile and water (UPLC grade) (1:10, v:v) with 1 mM ammonium acetate and pH was adjusted to 4.15 with acetic acid. Mobile phase B consisted of acetonitrile and 2-propanol (1:1, v:v). The liquid chromatography condition was adopted from a previous publication.^6^ In brief, the gradient was started with 90% A at an initial flow rate of 0.6 mL/min for 0.1 min, followed by a linear reduction of A to 65% from 0.1 to 9.25 min and another further reduction to 15% from 9.25 to 11.5 min. Between 11.5 and 11.8 min, the flow rate was increased to 0.65 mL/min and the solvent A was reduced from 15% to 0%. Between 11.8 to 12.4 min, 100% B was used to wash off the lipidic matrix at a flow rate of 0.8-1 mL/min. Between 12.45 and 15 min, the column was conditioned to the initial condition and the flow rate was reduced to 0.6 mL/min. The MS system was equipped with an electrospray ionization source operating in negative ion mode (ESI−). Mass spectrometry parameters were as follows: capillary voltage was set at 1.5 kV, cone voltage at 60 V, source temperature at 150 °C, desolvation temperature at 600 °C, desolvation gas flow at 1000 L/h, and cone gas flow rate at 150 L/h.

Seven injections of solvent blanks and 10 injections of the QC sample were carried out before the analysis of the samples. The QC sample was injected once every 11 sample injections. Thirteen mixtures of bile acid standards were injected at the end of the sample run for identification. Automatic data dependent acquisition (DDA), MS^E^ and dynamic range enhancement (DRE) MS methods were applied to the QC sample for bile acid identification.

The raw data were converted to NetCDF format using Databridge built in MassLynx V4.1 (Waters, Inc) and the data were extracted using XCMS package in R software. The PCA and OPLS-DA analysis were performed using SIMCA 14 (Umetrics, Sweden). Kruskal-Wallis test was used to test significance of the relative concentrations of bile acids observed amongst different time points. Dunn’s test was used for adjusting multiple comparisons.

**DNA extraction of fecal samples, 16S rRNA gene-based sequencing and data analysis**

DNA was extracted from 250 mg fecal samples using a modified protocol based on the Qiagen Stool Kit (Qiagen, Crawly, UK) with an additional bead beating step to homogenize and lyse bacteria in the samples (0.1 g 0.1mm sterile glass beads, 3 × 4500 rpm for 30 secs with 5 mins on ice in between cycles). DNA obtained from this extraction was quantified using the Invitrogen Qubit platform and diluted to a working concentration of 10 ng·μL^−1^. The polymerase chain reaction (PCR) was used to amplify the V1-V3 regions of the 16S rRNA gene from each DNA sample. The PCR was performed in triplicate on all DNA extracts using a MJ Research PTC-200P Thermal Cycler (MJ Research, USA). PCR mixtures (25 μL) contained 1 X Buffer (20 mM Tris pH 8.4, 50 mM KCl), 1.5 mM MgCl2, 200 μM of each dNTP, 1.25 U of Taq polymerase (NEB, UK), 5 pmol of each primer and 10 ng of DNA. The PCR conditions were: 95°C for 5 min initial denaturation, followed by 25 cycles of amplification at 95°C denaturation for 30 s, annealing at 55°C for 40 s and extension of 72°C for 1 min, with a final extension of 72°C for 5 min. PCR products were pooled for each sample, purified using a Qiagen PCR purification kit, quantified and equimolar amounts pooled prior to 454 pyrosequencing.

DNA sequencing was undertaken by Research and Testing Laboratory (Austin, Tex) using the Roche 454 pyrosequencer. The sequences were binned according to their sample source and processed via the RDP’s pyropipeline to remove any reads that were less than 250 bp and which contained any ambiguities. The filtered sequences were classified using the RDP classifier and the relative proportions of phyla and families determined. Community analysis of the data was undertaken using MOTHUR. The bacterial abundance data was analyzed using MicrobiomeAnalysis.^7,8^ Bacterial genera with <5 counts in all samples, <10% prevalence or <10% inter-quantile range were excluded. Kruskal-Wallis test was used to test significance of the relative abundances of bacterial genera amongst different time points. Dunn’s test was used for adjusting multiple comparisons.

**Fecal batch culture and bacterial isolate culture**

Feces were collected from two RYGB patients at 2-year post-op from Hammersmith Hospital, London, UK (ethics reference 13/LO/1510) and two healthy donors at St. Mary’s Hospital, London, UK (ethics reference 13/LO/1867). Patient fecal samples were mixed with 8% sterile dimethyl sulfoxide (DMSO) as cryoprotectant and stored at -80°C. Health donor samples were homogenized under anaerobic conditions and used fresh.

Batch cultures of fecal samples were cultured in YCFA medium supplemented with L-tyrosine, L-phenylalanine or L-tryptophan. A liter of YCFA broth was prepared and cooled to ≤50°C. Three beakers of 200 mL of YCFA media were supplemented with the corresponding amounts of aromatic amino acids (0.33 g of L-phenylalanine (10 mM) to beaker 1, 0.408 g of L-tryptophan (10 mM) to beaker 2 and 0.09 g of L-tyrosine (2.5 mM) to beaker 3) prior to being filter-sterilized and aliquoted into 50 mL sterile falcon tubes for degassing in an anaerobic chamber overnight.

A 10% fecal slurry (w/v) sample was obtained by mixing 3 g of the feces with 30 mL of 10% PBS and was homogenized on a vortex mixer until no clumps remained, and the debris was allowed to settle. An amount of 1.2 mL of the 10% fecal slurry was inoculated in a falcon tube containing 10.8 mL of the media prepared above. The batch culture was carried out anaerobically in triplications at each time point (i.e. 0, 7, 24 and 48 hours). Media were collected by centrifugating the tubes at 10,000 *g* to pellet the debris and filtered through a filter with a pore size of 0.2 µm before storing at -80°C pending for ^1^H NMR analysis.

Twelve representative bacterial species in healthy individuals (*Bacteroides uniformis* and *B. vulgatus*), obese patients (*Streptococcus cristatus*, *S. gallolyticus*, *Ruminococcus torques* and *Dorea longicatena*) and RYGB patients (*Shigella sonnei*, *Klebsiella pneumoniae*, *Escherichia coli*, *Enterobacter hormaechei*, *E. cloacae* and *K. oxytoca*) were selected for batch culture. *B. uniformis*, *B. vulgatus*, *R. torques* and *D. longicatena* were previously isolated from stool donated by a healthy 29-year-old male. *S. cristatus* (LMG 14512), *S. gallolyticus* (LMG14619), *S. sonnei* (LMG 10473)*, K. pneumonia* (LMG 3079)*, E. coli* (LMG 2092)*, E. hormaechei* (LMG 30171)*, E. cloacae* (LMG 2783) and *K. oxytoca* (LMG 3055) were purchased from UGent Laboratory of Microbiology (BCCM, Gent, Belgium). A defined simple bacterial culture medium was prepared based on a previous publication,^9^ supplemented with L-tryptophan, L-phenylalanine, L-tyrosine and choline. The media was de-gassed with N_2_ to create an anaerobic environment. The bacteria were cultured anaerobically in an anaerobic chamber for 24 hours and the media were collected using the method described above for ^1^H NMR analysis.

Sample preparation and ^1^H NMR analysis of medium samples were the same as the urinary samples mentioned above.

**Figure S1** ROC analysis of cross-validated scores from OPLS-DA models. (A) NMR analysis of urine; Tcv1 and Tcv2 represent the first and second PLS component when 2 PLS components were applied to separate 3 classes; (B) LC-MS analysis of urine; (C) bile acid analysis of urine; (D) NMR analysis of serum; (E) NMR analysis of feces.

**Figure S2** Relative metabolite levels in urine from cohorts 1 (A) and 2 (B), plasma from cohorts 3 (C) and 2 (D), and feces from cohort 1 (E). Metabolite levels are indicated by relative peak heights from the median fold normalized spectra of urine and feces and non-normalized serum spectra. Error bars are presented in SEM. a.u. stands for arbitrary unit.

**Figure S3** OPLS-DA cross-validated scores plots of urinary ^1^H NMR spectra of the RYGB and LGB patients from cohort 3 at pre-op (Q^2^Y=0.49; R^2^X=12.35%; R^2^Y=89.6%; p= 0.006), and 6-month post-op (Q^2^Y=0.44; R^2^X=30.66%; R^2^Y=95.2%; p=0.038). The metabolites that significantly contributed to the classification of different time points are shown in Figure 2C.

**Figure S4** OPLS-DA cross-validated scores plots of urinary reverse-phase liquid chromatography-mass spectrometry profiles of the bariatric patients from cohort 1 in ESI positive mode (Q^2^Y=0.46; R^2^X=24.8%; R^2^Y=73.4%; CVANOVA p = 2.9 x 10^-14^) and ESI negative mode (Q^2^Y=0.52; R^2^X=14%; R^2^Y=76.6%; CVANOVA p = 6.2 x 10^-17^). The identified metabolites that significantly contributed to the classification between pre-op (black) and 2-6 months post-op (green) are listed in the table. Positive correlation (r) represents higher relative concentrations of these metabolites at the post-op compared to pre-op. p[1] is loadings from the OPLS-DA models. Coefficient of variation (CV) was calculated based on the quality control (QC) samples to evaluate the analytical variation of the features.

**Figure S5** OPLS-DA cross-validated scores plots of urinary bile acid profiles of the RYGB patients from cohort 1 at pre-op (black), 2-6 months (green) and 1-2 years (orange) post-op (A. Q^2^Y=0.21; R^2^X=16.9%; R^2^Y=48.9%; CVANOVA p= 5.8x10^-9^). Bar plots of relative intensities of urinary bile acids identified from cohort 2 RYGB patients at pre-op (black) and 6 months post-op (green). Wilcoxon matched pairs signed rank test was used. ** 0.001<p<0.01. Data were presented in mean±SEM. The bile acids and their retention time (min) and m/z were given in the titles.

**Figure S6** Relative intensities of urinary bile acids from sleeve gastrectomy (SG) and laparoscopic gastric banding (LGB) patients from cohort 1 at pre-op (black), 2-6 months (green) and 1-2 years post-op (orange). Kruskal-Wallis test was used, and Dunn’s test was used for adjusting multiple comparisons. The adjusted p values: ****, p<0.0001, ***, p<0.001, ** p<0.01, * p<0.05. Data were presented in mean±SEM. The bile acids and their retention time (min) and m/z were given in the titles.

**Figure S7** OPLS-DA cross-validated scores plots of serum ^1^H NMR CPMG spectra of the RYGB patients from cohort 3 between pre-op and 3 (A, Q^2^Y=0.69; R^2^X=35.1%; R^2^Y=98.2% p= 0.002), 6 (B, Q^2^Y=0.63; R^2^X=40.4%; R^2^Y=97.9% p= 0.002), 9 (C, Q^2^Y=0.80; R^2^X=37.9%; R^2^Y=98.6% p= 0.002) or 12-month (D, Q^2^Y=0.68; R^2^X=45.5%; R^2^Y=98.3% p= 0.008) post-op. OPLS-DA cross-validated scores plots of serum ^1^H NMR CPMG spectra of the RYGB patients between pre-op and 6-month post-op of cohort 2 (E, Q^2^Y=0.56; R^2^X=37.7%; R^2^Y=87.7% p= 0.002) or combined cohorts 2 and 3 (F, Q^2^Y=0.25; R^2^X=31.4%; R^2^Y=72.9% p= 0.002).

**Figure S8.** Partial ^1^H NMR spectra of faecal batch culture media supplemented with tyrosine or tryptophan at 0 (black), 7 (blue), 24 (red) and 48 (pink) hours. Triplicates were carried out and the median spectra at each time point are shown. The top and middle panels are from healthy and RYGB donors, respectively. The bottom panel is from one of the RYGB donors. X axis is chemical shift in ppm and Y axis is peak intensities.

 **Figure S9.** Partial ^1^H NMR spectra of bacterial isolate culture in a defined simple media supplemented with phenylalanine (top panel), tryptophan (middle panel) or choline (bottom panel). Triplicates were carried out and all media spectra are shown. Black: control media; blue: *B. uniformis*; red: *K. oxytoca,* pink: *E. cloacae; green: E. coli*; light blue: *B. vulgatus.* X axis is chemical shift in ppm and Y axis is peak intensities.

**Table S1: Summary of exemplar human clinical studies investigating the metabolic effects of bariatric surgery to date.**

| **Study description** | **No.** | **Time points** | **Biofluids, (platform) & metabolic observations** |
| --- | --- | --- | --- |
| Roux-en-Y Gastric Bypass Surgery | 26 | 15 & 90 days post | ^10^Dried blood spots (FIA-MS): increased products of fatty acid (FA) catabolism- acylcarnitines (acetylcarnitine (C2), long-chain AC species -mainly C16 and C18), sphingomyelins15 days after surgery hydroxybutyrylcarnitine (C4-OH) and. Lower propionylcarnitine (C3) 15 &90 days post. Approx 50% increase in bile acids - glycochenodeoxycholic acid, glycocholic acid & taurochenodeoxycholic acid |
|  | 20 | 14 D & 6 M post | ^11^Serum (NMR & GC-MS): non-esterified fatty acids, branched chain amino acids, 2-aminobutyrate, butyrate, 2-hydroxybutyrate, 3-hydroxybutyrate, acetone, 2-methylglutarate, and 2-oxoisocaproate reduced at 6M. Increased alanine, glycine, pyruvate, taurine & FA (C10:0, C13:0, C14:0, C15:0, and C18:0) at 6M PS. |
|  | 9 | Pre-, 3M post | ^12^Serum (GC-MS) RYGB increases caseinate digestion and amino acid absorption leading to high transient postprandial increase in plasma amino acids. |
|  | 20 | Pre- post | ^13^Serum (UPLC-MS, GC-MS) reduced levels glucose, pyruvate, lactate. BCAAs, kyurenine, kyurenate, palmitate, stearate, and oleate, hypoxanthine, xanthine, urate, and allantoin increased 3-indoxylsulfate, adenosine and inosine |
|  | 16 | Pre-, 3W, 3M post | ^14^Plasma (LC-MS): decrease in BCAAS |
|  | 14 | Pre-, 3M, 6M post | ^15^Serum (LC-MS & GC-MS): increased p-cresol sulfate, galactose, phosphatidylcholine and decrease tyrosine, kynurenic acid, threonine,lysine, BCAAs,pentadecanol, stearic acid, ceramide |
|  | 10 | Pre- & 12M post | ^16^Serum (NMR): decreased fasted lactate, alanine & BCaas post surgery; decreased very low-density lipoprotein, low-density lipoprotein, N-acetyl-glycoproteins, and unsaturated lipid levels; increased phosphatidylcholine and high-density lipoprotein |
|  | 10 | Pre-, 12M post | ^17^Plasma (NMR & GC-MS) decrease in lactate, leucine, isoleucine, valine, lactate, glucose , lipoprotein, unsaturated lipids, and N-acetyl-glycoprotein were higher before RYGB high-density lipoprotein and phosphatidylcholine were higher after bariatric surgery |
|  | 44 | Pre, 2W & 12M post | ^18,19^Serum (LC-MS): increased trimethylamine-*N*-oxide, indoxyl sulphate; reduced alanine, choline, BCAA, phenylalanine PS. Decrease in alanine after one year in the group of patients with diabetes remission relative to non-remission |
|  | 18 | Pre, 1W & 3M post | ^19^Serum (GC- & LC-MS): Increased conjugated bile acids (glyoxylate, glychochenodeoxycholic acid, taurochenodeoxycholic), p-cresol, 3-indoxylsulfuric, indole3-propionic; Decreased – myristic & 2-hydroxybutyrate. Leptin is strongly associated with amino acids (leucine and tryptophan); IL-6 is independent of carbohydrates and amino acids but correlates with complex fatty acids and lipids (ceramide & palmitic acid). |
| Sleeve Gastrectomy | 32 | Pre-, 6M post | ^20^serum (LC-MS) Increase in polyamine metabolism, e.g. putrescine and acetyl derivatives of spermidine and spermine after bariatric surgery. |
|  | 39 | Pre- & 6M post | ^21^Serum (GC- & LC-MS): waist circumference &cholesterol levels correlated with multiple lipids; hexoses correlated with glycaemic levels. |
|  | 8 | Pre & 6M post | ^22^Blood (LC-MS): decrease in fasting phenylalanine, glutamine/glutamate, and tyrosine concentrations, BCAAs, alanine, glutamate/glutamine, methionine, phenylalanine. |
| Metabolic & micobiome changes post bariatric surgery | 61 | Pre-, 1,3 & 12 M post | ^23^Serum (GC- & LC-MS): glycine, acetylglycine, methylmalonate increased post-RYGB negatively correlated with the decrease in body corpulence. At 12M PS Acetylglycine and glycine increased and were negatively associated with improved weight and body composition.  *3-Methoxyphenylacetic acid*, positively associated with microbial gene richness & negatively with trunk-fat mass. Low gene richness (LGC) correlated with increased trunk-fat mass and comorbidities (type 2 diabetes, hypertension and severity). *Hungatella hathewayi*, *Fusobacterium nucleatum* and *GU:588 Dialister sp* had strongest negative associations with BMI. |
|  | 23 | Pre, 1 &3M post | ^24^Serum (GC- & LC-MS): decreased aromatic amino acids & glutamate; increased methionine, alanine & lysine. Increased *B. thetaiotaomicron* |
|  | 26 | Pre-, 3M, 6\|M, 12M post | ^25^Plasma (LC-MS) increased circulating TMAO, choline and bile acids PS.  Increased alpha diversity, and altered beta diversity composition and function up to 6 months after surgery (regressing to presurgery levels at 12M). 12 enriched bacterial pathways at 6M regressing by 12M |
| Diabetes remission after bariatric surgery | 20 | Pre & 1M post | ^26^Serum (GC- & LC-MS): increase in phospho- & lysophosphocholines; decrease in phosphoethanolamine & arachadonic acid acid metabolites. Lactate, oleic acid, androsterones decreased in fast responders |
|  | 35 | Pre, 6M & 12M post | ^27^Serum (LC-MS): Baseline levels of tryptophan, bilirubin, and indoxyl sulfate (higher in remission group), FFA 16:0, FFA 18:3, FFA 17:2 measured prior to surgery as well as levels and hippuric acid (lower in remission group) measured at 6 months after surgery best predicted the suitability and efficacy of RYGB for patients with T2DM |
|  | 15 | Pre-, 14, 20 days post | ^28^Serum (LC-MS): Histidine derivatives; glutathione and its precursors were more abundant following weight-loss surgery. Decrease in urate, ascorbic acid and increase in hypoxanthine and allantoin |
|  | 38 | Pre, 12M, 24M | ^29^Serum (LC-MS): higher baseline stearic acid/palmitic acid (S/P) ratio associated with T2D remission post RYGB. 68.4% demonstrated diabetes remission at 24M post surgery. At 1 yr after RYGB ratios of eicosatrienoic acid/ eicosadienoic acid (C20:3 n6/C20:2 n6) and eicosatrienoic acid/g-linolenic acid (C20:3 n6/C18:3n6) were increased whereas a-linolenic acid/linoleic acid and g-linolenic acid/linoleic acid ratios decreased. |
|  | 11 | Pre- & 6M post | ^30^Plasma (GC-MS): decreased 2-hydroxybutyric acid & glycine; increased nonesterified fatty acids (NEFA), valine, BCAA (combined leucine/isoleucine and valine), and glutamate/glutamine |
|  | 16 | Pre, 4D, 42D post | ^31^Plasma (LC-MS) Increased decanoic acid and octanoic acid, decreased sphingomyelins. Participants in T2D remission had higher pre-surgery levels of tricarboxylic acid cycle intermediates and triglycerides with long-chain fatty acids compared with subjects not in remission. |
| sustained weight loss versus regain post RYGB | 35 | >6M post | ^31^Serum (LC-MS)lower serum levels of metabolites related to the serine, glycine and threonine pathway, phenylalanine metabolism, tricyclic acid cycle, alanine and glutamate metabolism, and higher levels of other amino acids. |
| Quick versus slow resolution of T2D after bariatric surgery | 20 | Pre-, 1M PS | ^26^Plasma (LC-MS) Lysophosphatidylcholines increase lysophosphatidylethanolamine and arachidonic acid decrease post surgery. Acetyl carnitines, oleamide, stearamide, choline, sphingosine piperine increase more in patients where T2D remission was quick, lactic, uric and oleic acids, lysophosphatidylcholines, lysophosphatidylethanolamine and phosphoethanolamices, taurine, androsterone decrease |
| Restrictive vs malabsorptive bariatric surgery | 37 | Pre & 6M PS | ^32^Serum (LC-MS): sleeve gastrectomy increased (lyso)PCs & (lyso)PEs, especially those with ether linkage & sphingolipids (ceramides & sphingomyelins). BMI positively correlated with an increase of 1-ether, 2-acyl phosphocholine (PC O2) species |
|  | 106 | Pre-, 3, 6, 9 & 12M PS | ^33^Serum (NMR): increased aromatic amino acids phenylalanine and tyrosine, and the BCAAs leucine, isoleucine, and valine, as well as reduced concentrations of glutamine and histidine PS. Temporary increase in citrate, pyruvate, acetoacetate, 3-hydroxybutyrate. Dimethylsulfone only increased in patients undergoing distal RYGB. TMAO increased after RYGB but not sleeve. |
|  | 2 | Pre-, 3M PS | ^34^Urine (NMR) increased hippurate, xanthine and trigonelline in both types of surgery |
|  | 54 | Pre-, 1M & 6M PS | ^26^Serum (GC- & LC-MS): 4-cresol increased post sleeve gastrectomy; phenol sulphate increased in RYGB at 6M post. Choline decreased in both groups at 1M PS |

Key : branched chain amino acid (BCAA); day (D); fatty acid (FA); gas chromatography mass spectrometry (GC-MS); liquid chromatography mass spectrometry (LC-MS); month (M); nuclear magnetic resonance (NMR); post-surgery (PS); Roux-en-Y Gastric bypass (RYGB); type 2 diabetes (T2D)

**Table S2** Summary of the number of patients and body mass index (BMI) at each time based on types of bariatric surgery. Values are shown as mean±SD. Kruskal-Wallis ANOVA test was used to test the significance of BMI changes across the time points in cohorts 1 and 3, while Friedman test was used for cohort 2 for paired analysis.

| **RYGB** | **Cohort 1** | | | **Cohort 3** | | | | | | **Cohort 2** | |
| --- | --- | --- | --- | --- | --- | --- | --- | --- | --- | --- | --- |
| **Time points** | **Pre-op** | **2-6 mo** | **12-24 mo** | **Pre-op** | **1 mo** | **3 mo** | **6 mo** | **9 mo** | **12 mo** | **Pre-op** | **6 mo** |
| **Patients (n)** | 68 | 58 | 48 | 9 | 6 | 8 | 7 | 7 | 4 | 9 | 9 |
| **Sex (Female:Male)** | 49:19 | 40:18 | 36:12 | 5:4 | 4:2 | 5:3 | 3:4 | 3:4 | 2:2 | 9:0 | 9:0 |
| **Age at the surgery (years)** | 43.1± 9.1 | | | 46.1±9.7 | | | | | | 39.00 ± 8.57 | |
| **BMI (kg/m^2^)** | 48.02±8.35 (n=57) | 38.81±9.07 (n=46) | 34.84±6.81 (n=38) | 48.99±9.99 (n=9) | 41.2±7.89 (n=6) | 37.47±6.53 (n=7) | 36.35±8.16 (n=7) | 33.64±7.86 (n=7) | 30.73±4.51 (n=4) | 44.73 ± 3.56 (n=9) | 32.20 ± 5.17 (n=11) |
| **P values for BMI across time points** | <0.0001 | | | 0.0054 | | | | | | <0.0001 | |

| **SG** | **Cohort 1** | | |
| --- | --- | --- | --- |
| **Time point** | **Pre-op** | **2-6 mo** | **12-24 mo** |
| **Number of patients (n)** | 10 | 7 | 4 |
| **Age at the surgery (years)** | 45.4 ± 9.0 | | |
| **Sex (Female:Male)** | 5/5 | 4/3 | 2/2 |
| **BMI (kg/m^2^)** | 54.82±10.63 (n=9) | 38.65±9.47 (n=4) | 38.25±4.16 (n=4) |
| **P values for BMI across time points** | 0.0108 | | |

| **LGB** | **Cohort 1** | | | **Cohort 2** | |
| --- | --- | --- | --- | --- | --- |
| **Time point** | **Pre-op** | **2-6 mo** | **12-24 mo** | **Pre-op** | **6 mo** |
| **Number of patients (n)** | 6 | 5 | 3 | 5 | 5 |
| **Age at the surgery (years)** | 55.4 ± 8.6 | | | 29.8 ± 9.1 | |
| **Sex (Female:Male)** | 5:1 | 5:0 | 3:0 | 5:0 | 5:0 |
| **BMI (kg/m^2^)** | 48.04 ± 5.62 (n=5) | 41.14 ±5.08 (n=5) | 40.40 ± 0.60  (n=3) | 40.50 ± 5.73 (n=5) | 35.15 ± 6.44 (n=5) |
| **P values for BMI across time points** | 0.0647 | | | <0.0001 | |

**References**

1 Dona AC, Jimenez B, Schafer H*, et al.* Precision high-throughput proton NMR spectroscopy of human urine, serum, and plasma for large-scale metabolic phenotyping. *Anal Chem*  2014;**86**:9887-94.

2 Gratton J, Phetcharaburanin J, Mullish BH*, et al.* Optimized Sample Handling Strategy for Metabolic Profiling of Human Feces. *Analytical chemistry*  2016;**88**:4661-8.

3 Veselkov KA, Lindon JC, Ebbels TM*, et al.* Recursive segment-wise peak alignment of biological (1)h NMR spectra for improved metabolic biomarker recovery. *Anal Chem*  2009;**81**:56-66.

4 Want EJ, Wilson ID, Gika H*, et al.* Global metabolic profiling procedures for urine using UPLC-MS. *Nature protocols*  2010;**5**:1005-18.

5 Veselkov KA, Vingara LK, Masson P*, et al.* Optimized Preprocessing of Ultra-Performance Liquid Chromatography/Mass Spectrometry Urinary Metabolic Profiles for Improved Information Recovery. *Analytical chemistry*  2011;**83**:5864-72.

6 Sarafian MH, Lewis MR, Pechlivanis A*, et al.* Bile acid profiling and quantification in biofluids using ultra-performance liquid chromatography tandem mass spectrometry. *Anal Chem*  2015;**87**:9662-70.

7 Chong J, Liu P, Zhou G*, et al.* Using MicrobiomeAnalyst for comprehensive statistical, functional, and meta-analysis of microbiome data. *Nat Protoc*  2020;**15**:799-821.

8 Dhariwal A, Chong J, Habib S*, et al.* MicrobiomeAnalyst: a web-based tool for comprehensive statistical, visual and meta-analysis of microbiome data. *Nucleic Acids Res*  2017;**45**:W180-W8.

9 Jameson E, Fu T, Brown IR*, et al.* Anaerobic choline metabolism in microcompartments promotes growth and swarming of Proteus mirabilis. *Environ Microbiol*  2016;**18**:2886-98.

10 Fiamoncini J, Fernandes Barbosa C, Arnoni Junior JR*, et al.* Roux-en-Y Gastric Bypass Surgery Induces Distinct but Frequently Transient Effects on Acylcarnitine, Bile Acid and Phospholipid Levels. *Metabolites*  2018;**8**.

11 Wijayatunga NN, Sams VG, Dawson JA*, et al.* Roux-en-Y gastric bypass surgery alters serum metabolites and fatty acids in patients with morbid obesity. *Diabetes Metab Res Rev*  2018;**34**:e3045.

12 Bojsen-Moller KN, Jacobsen SH, Dirksen C*, et al.* Accelerated protein digestion and amino acid absorption after Roux-en-Y gastric bypass. *Am J Clin Nutr*  2015;**102**:600-7.

13 Modesitt SC, Hallowell PT, Slack-Davis JK*, et al.* Women at extreme risk for obesity-related carcinogenesis: Baseline endometrial pathology and impact of bariatric surgery on weight, metabolic profiles and quality of life. *Gynecol Oncol*  2015;**138**:238-45.

14 Lips MA, Van Klinken JB, van Harmelen V*, et al.* Roux-en-Y gastric bypass surgery, but not calorie restriction, reduces plasma branched-chain amino acids in obese women independent of weight loss or the presence of type 2 diabetes. *Diabetes Care*  2014;**37**:3150-6.

15 Mutch DM, Fuhrmann JC, Rein D*, et al.* Metabolite profiling identifies candidate markers reflecting the clinical adaptations associated with Roux-en-Y gastric bypass surgery. *PLoS One*  2009;**4**:e7905.

16 Lopes TI, Geloneze B, Pareja JC*, et al.* "Omics" Prospective Monitoring of Bariatric Surgery: Roux-En-Y Gastric Bypass Outcomes Using Mixed-Meal Tolerance Test and Time-Resolved (1)H NMR-Based Metabolomics. *OMICS*  2016;**20**:415-23.

17 Lopes TI, Geloneze B, Pareja JC*, et al.* Blood Metabolome Changes Before and After Bariatric Surgery: A (1)H NMR-Based Clinical Investigation. *OMICS*  2015;**19**:318-27.

18 Narath SH, Mautner SI, Svehlikova E*, et al.* An Untargeted Metabolomics Approach to Characterize Short-Term and Long-Term Metabolic Changes after Bariatric Surgery. *PLoS One*  2016;**11**:e0161425.

19 Seridi L, Leo GC, Dohm GL*, et al.* Time course metabolome of Roux-en-Y gastric bypass confirms correlation between leptin, body weight and the microbiome. *PLoS One*  2018;**13**:e0198156.

20 Ocana-Wilhelmi L, Cardona F, Garrido-Sanchez L*, et al.* Change in serum polyamine metabolome pattern after bariatric surgery in obese patients with metabolic syndrome. *Surg Obes Relat Dis*  2020;**16**:306-11.

21 Palau-Rodriguez M, Tulipani S, Marco-Ramell A*, et al.* Characterization of Metabolomic Profile Associated with Metabolic Improvement after Bariatric Surgery in Subjects with Morbid Obesity. *J Proteome Res*  2018;**17**:2704-14.

22 Yao J, Kovalik JP, Lai OF*, et al.* Comprehensive Assessment of the Effects of Sleeve Gastrectomy on Glucose, Lipid, and Amino Acid Metabolism in Asian Individuals with Morbid Obesity. *Obes Surg*  2019;**29**:149-58.

23 Aron-Wisnewsky J, Prifti E, Belda E*, et al.* Major microbiota dysbiosis in severe obesity: fate after bariatric surgery. *Gut*  2019;**68**:70-82.

24 Liu R, Hong J, Xu X*, et al.* Gut microbiome and serum metabolome alterations in obesity and after weight-loss intervention. *Nat Med*  2017;**23**:859-68.

25 Shen N, Caixas A, Ahlers M*, et al.* Longitudinal changes of microbiome composition and microbial metabolomics after surgical weight loss in individuals with obesity. *Surg Obes Relat Dis*  2019;**15**:1367-73.

26 Samczuk P, Hady HR, Adamska-Patruno E*, et al.* In-and-Out Molecular Changes Linked to the Type 2 Diabetes Remission after Bariatric Surgery: An Influence of Gut Microbes on Mitochondria Metabolism. *Int J Mol Sci*  2018;**19**.

27 Luo P, Yu H, Zhao X*, et al.* Metabolomics Study of Roux-en-Y Gastric Bypass Surgery (RYGB) to Treat Type 2 Diabetes Patients Based on Ultraperformance Liquid Chromatography-Mass Spectrometry. *J Proteome Res*  2016;**15**:1288-99.

28 Sarosiek K, Pappan KL, Gandhi AV*, et al.* Conserved Metabolic Changes in Nondiabetic and Type 2 Diabetic Bariatric Surgery Patients: Global Metabolomic Pilot Study. *J Diabetes Res*  2016;**2016**:3467403.

29 Zhao L, Ni Y, Yu H*, et al.* Serum stearic acid/palmitic acid ratio as a potential predictor of diabetes remission after Roux-en-Y gastric bypass in obesity. *FASEB J*  2017;**31**:1449-60.

30 Shantavasinkul PC, Muehlbauer MJ, Bain JR*, et al.* Improvement in insulin resistance after gastric bypass surgery is correlated with a decline in plasma 2-hydroxybutyric acid. *Surg Obes Relat Dis*  2018;**14**:1126-32.

31 Arora T, Velagapudi V, Pournaras DJ*, et al.* Roux-en-Y Gastric Bypass Surgery Induces Early Plasma Metabolomic and Lipidomic Alterations in Humans Associated with Diabetes Remission. *PLoS One*  2015;**10**:e0126401.

32 Ramos-Molina B, Castellano-Castillo D, Alcaide-Torres J*, et al.* Differential effects of restrictive and malabsorptive bariatric surgery procedures on the serum lipidome in obese subjects. *J Clin Lipidol*  2018;**12**:1502-12.

33 Gralka E, Luchinat C, Tenori L*, et al.* Metabolomic fingerprint of severe obesity is dynamically affected by bariatric surgery in a procedure-dependent manner. *Am J Clin Nutr*  2015;**102**:1313-22.

34 Calvani R, Miccheli A, Capuani G*, et al.* Gut microbiome-derived metabolites characterize a peculiar obese urinary metabotype. *Int J Obes (Lond)*  2010;**34**:1095-8.
